# Vessel noise affects routine swimming and escape response of a coral reef fish

**DOI:** 10.1371/journal.pone.0235742

**Published:** 2020-07-23

**Authors:** Laura Velasquez Jimenez, Eric P. Fakan, Mark I. McCormick

**Affiliations:** Department of Marine Biology and Aquaculture, ARC Centre of Excellence for Coral Reef Studies, James Cook University, Townsville, Queensland, Australia; Swansea University, UNITED KINGDOM

## Abstract

An increasing number of studies have shown that anthropogenic noise can negatively affect aspects of the anti-predator behaviour of reef fishes, potentially affecting fitness and survival. However, it has been suggested that effects could differ among noise sources. The present study compared two common sources of anthropogenic noise and investigated its effects on behavioural traits critical for fish survival. In a tank-based experiment we examined the effects of noise from 4-stroke motorboats and ships (bulk carriers > 50,000 tonnes) on the routine swimming and escape response of a coral reef fish, the whitetail damselfish (*Pomacentrus chrysurus*). Both 4-stroke boat and ship noise playbacks affected the fast-start response and routine swimming of whitetail damselfish, however the magnitude of the effects differed. Fish exposed to ship noise moved shorter distances and responded more slowly (higher response latency) to the startle stimulus compared to individuals under the 4-stroke noise treatment. Our study suggests that 4-stroke and ship noise can affect activity and escape response of individuals to a simulated predation threat, potentially compromising their anti-predator behaviour.

## Introduction

Human activities are prevalent throughout marine environments. These activities contribute significant amounts of noise to marine soundscapes, thereby increasing overall ambient sound levels [1–3]. Recreational boating and commercial shipping are two of the most common sources of anthropogenic noise in marine ecosystems, particularly those found along the Australian coast [4]. In 2014, there were 90,000 recreational motorboats registered in Queensland and 9,619 ships transited through the Great Barrier Reef. These numbers are projected to increase by 500 per cent and 250 per cent respectively by 2040 [5]. Currently, the management of underwater noise in Australia is still in its early stages compared to international underwater noise regulations [6], which is partly due to a global lack of supporting scientific evidence. Therefore, research on the effects of vessel noise on marine organisms is required to develop effective management policies.

Sound is one of the most important sensory cues used by fishes to obtain information about their environment [7]. Fish use sound for predator avoidance, communication, navigation, orientation, reproduction and feeding [7]. Vessel noise can interfere with these functions in a number of ways, including acting as a distracting stimulus [8], as a stressor [9–11], or by masking important acoustic cues or signals [12, 13]. Recent studies have found that interference with the ecological functions of fish by anthropogenic noise results in negative effects on the physiology and behaviour of at least some organisms regardless their life stage [14–16]. Therefore, anthropogenic noise is compromising the important role sound plays as an information source in marine ecosystems.

Distraction can have a critical effect on the outcome of predator-prey interactions [17]. In a predator-prey scenario, the escape response of aquatic prey is usually represented as sudden changes in direction and acceleration as a consequence of a startle stimulus (i.e., predator strike) [18]. Survival under natural conditions has been shown to be directly related to the speed of initiating an escape response (known as latency) [19]. Exposure to noise can affect the escape performance of individuals as a distracted prey may be less likely to respond to predators, which may reduce its likelihood of survival [14, 17]. Moreover, since most marine fishes have complex life histories involving a dispersive larval phase and a reef-associated adult phase, any factor that influences mortality at critical life stages [20] such as metamorphosis and settlement, can lead to significant changes in the numbers reaching the next life stage [21, 22].

This study presents results of a tank-based experiment that examined the routine swimming and detailed escape response of a juvenile reef fish, while investigating the effects of 4-stroke boat and ship noise in a controlled acoustic environment. Caution is needed when extrapolating results into the wild, as the use of tanks and speakers can result in sound fields that differ from the ones experienced by organisms in their natural environment [23, 24]. However, tank-based experiments allow for the control of confounding factors and detail data collection [25, 26], and can contribute significantly to the understanding of anthropogenic noise [17, 27, 28]. In our case it was possible to recreate a predator strike that was consistent over trials and examined in detail the effects of ship noise on behavioural traits critical for fish survival [19]. Recent research suggests that the effects of anthropogenic noise and their magnitude can vary according to the source due to differences in their acoustic characteristics (e.g. frequency and power spectra) [25, 29, 30]. Therefore, our hypothesis was that ship playback would be more detrimental than 4-stroke motorboats playback due to differences in the sounds produced.

## Materials and methods

### Study species and maintenance

The whitetail damselfish, *Pomacentrus chrysurus*, is a common coral reef fish found throughout the Indo Pacific region. Typically found in shallow reef waters (<10 m), it has a bipartite life history with a planktonic larval stage maintained for 20–25 days before individuals settle onto a coral reef [31, 32]. Previous research has shown that the hearing range of recently settled damselfishes is between 30 and 1000 Hz [2, 29, 30]. Recently settled *P*. *chrysurus* juveniles (12.09 mm mean standard length) were collected overnight using light traps around Lizard Island Research Station (14.6680° S, 145.4638° E) and transported to the research station in 60 L tanks. Fish were identified to species and placed in 30 L tanks for two days to acclimate. The flow-through seawater intake was placed below the surface to reduce ambient noise and no air-stone was present. Fish were fed twice a day with *Artemia spp*. Individuals were isolated and not fed for 24 hours prior to experimental trials in order to standardise for satiation.

All methods and research within this study were carried out in accordance with the animal ethics guidelines and regulations of James Cook University, and all protocols were approved by the James Cook University Animal Ethics Committee (approval number: A2408).

### Soundscapes

Three different acoustic stimuli were used as treatments: ambient sound, 4-stroke motorboat and ship noise. Three recordings were made of each acoustic stimulus (9 in total) during the day time at different locations around Lizard Island Research Station (see S1 Table for details). The ambient reef recordings were collected on healthy reefs between 6 to 9 m depth. The 4-stroke boat recordings were collected from three different research station boats (5 m long aluminium hulls, Suzuki 4-stroke 30 hp DF30A, engine power 22.1 kW) travelling at a near constant speed at distances ranging from 5 to 25 m from the hydrophone. The ship recordings were made from different passing ships (~53,000 tonne bulk carriers, engines type MAN-B&W Diesel; engine power 13,501 kW) at distances ranging from 1.9 to 3.0 km from hydrophone. All sound recordings were made using SoundTrap 202 (Ocean Instruments, New Zealand) digital sound recorders with a 48 ksps sample rate (manufacturer’s specifications of a flat response within ±3 dB between 20 Hz and 60 kHz).

Playback treatments were created from the field recordings using Audacity^™^ 2.2.1. For each of the acoustic treatments three playback tracks were created. All tracks were 15 minutes in duration. Each playback consisted of 10 minutes of ambient playback followed by 5 minutes of the respective treatment (continued ambient playback, 4-stroke motorboat or ship noise). For the ambient playbacks a random section of the ambient reef recordings was used; for 4-stroke motorboat and ship playbacks, the chosen 5 minutes were from the maximum amplitude period of the replicate recording.

The 370 L acoustic treatment tank (50 x 65 x 115 cm) was positioned on top of bricks, with a 4 cm layer of foam between the base of the tank and the bricks to reduce acoustic artefacts caused by vibration transfer. Acoustic treatments were played using a J9 underwater speaker. J9 speakers are able to reproduce low frequency energy (i.e. peak spectral levels in the frequency band 10-50Hz) [33]. The J9 was kept in a fixed position by a bungee cable attached to a wood structure at one side of the tank. Water depth was 50 cm and the speaker was suspended 15 cm below the surface. Sound treatments were played placing the speaker 15 cm away from the experimental arena. The sound system used for playback of the treatments consisted of a 12v battery, MP3 player (SanDisk 8gb Clip Jam), amplifier (18 W, Kemo Langen Germany) and a J9 speaker (NUWC-USRD, Newport, RI, USA).

Prior to the experiment, acoustic tank dynamics were investigated in a 4x3 grid pattern (S1 Fig). A calibrated tri-axial accelerometer (working frequency range 10–2000 Hz, [M20-040 from Geospectrum Technologies Inc, Dartmouth, Canada]) coupled to a Zoom F8 Multi-Track Field Recorder (Zoom Corporation) with a 96 kHz sampling rate was used to do independently measure particle acceleration and sound pressure at each position on the grid. The accelerometer was suspended from a series of vertical and horizontal beams to allow the sensor to be repositioned around the grid, maintaining a depth of 15 cm below the surface. Playback of sound, using ambient reef sound, 4-stroke noise and ship noise recordings was repeated for each of the grid circles. Sound analysis showed that the closest position central to the J9 speaker provided the most comparable sound pressure levels to the recordings from the field (S2–S5 Figs). As a result, the experimental arena was located in this position for subsequent trials.

### Experimental protocol

**Fig 1:**
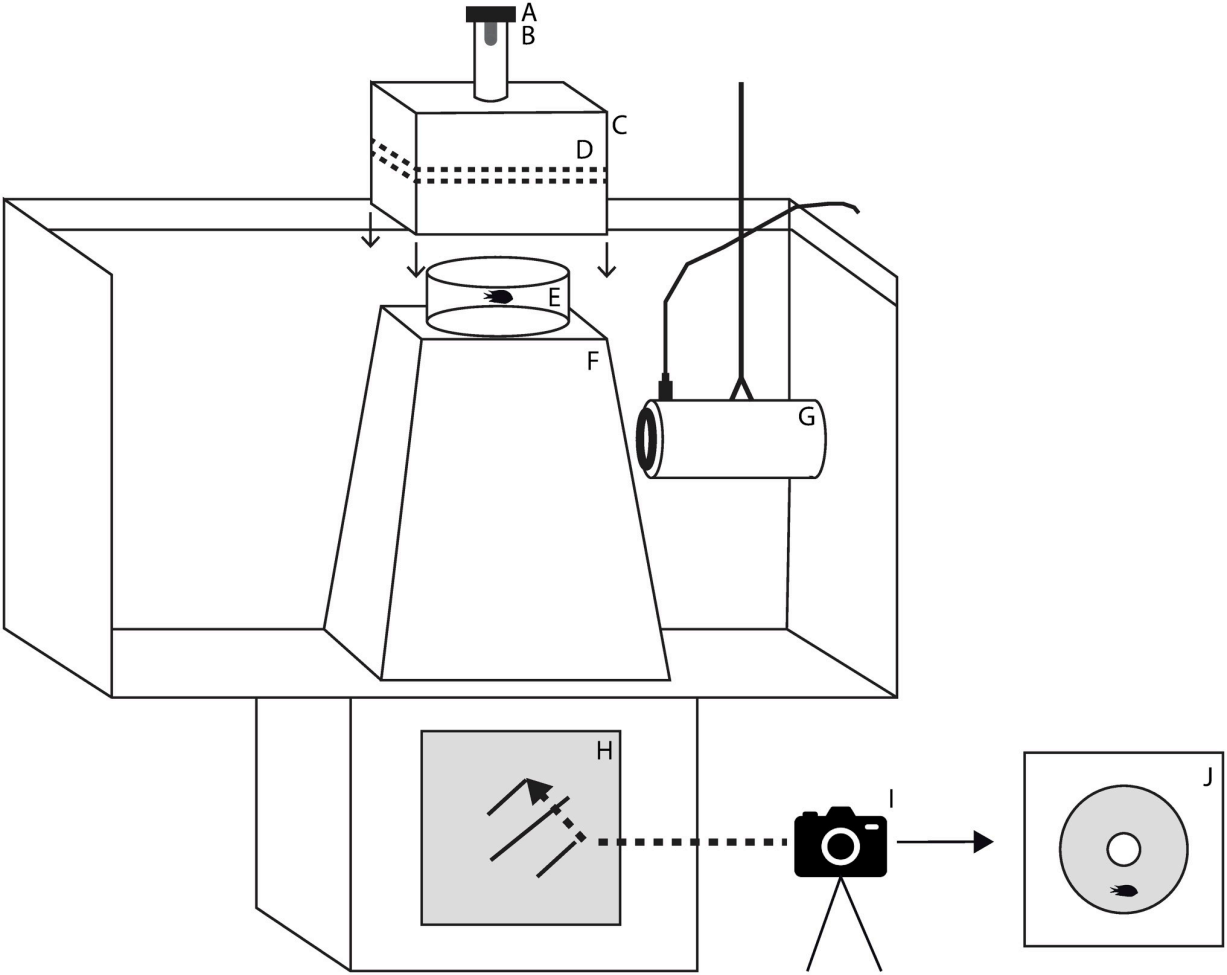
Schematic of the experimental set up. Experimental arena for analysis of routine swimming and escape response of *Pomacentrus chrysurus*. Electromagnet (A), tapered weight (B), opaque white container (C), LED lights (D), experimental arena (E), base of the experimental arena (F), J9 speaker (G), mirror (H), camera (I) and image projected from the mirror (J).

Individuals were carefully transferred using a jar from an acclimation tank into a circular experimental arena (diameter 20 cm; water level 4 cm) that was positioned inside a 370 L rectangular tank (50 x 65 x 115 cm). To minimise disturbance, the area above and around the arena was covered with an opaque white container to avoid laboratory disturbances and illuminated with LED lights (Fig 1). Water temperature during the experiment averaged 29.5°C.

Randomly selected individuals were exposed to one of three treatments: ambient playback (n = 27), 4-stroke boat noise playback (n = 26), or ship noise playback (n = 26). Each individual was tested only once. Individuals were given nine minutes to habituate to the experimental arena. Routine swimming and escape response were recorded from below the tank with a camera (Casio EX-ZR1000) at 30 frames per second and 480 frames per second, respectively. Video recordings were later analysed using ImageJ software (https://imagej.nih.gov/ij/). Video analysis was based on the centre of mass of the fish, the point about which propulsive forces act [34]. To avoid observer bias, the video and audio recordings were analysed blind to the sound treatment. During the trials, individuals did not startle when either 4-stroke or ship noise started.

#### Routine swimming

**Fig 2:**
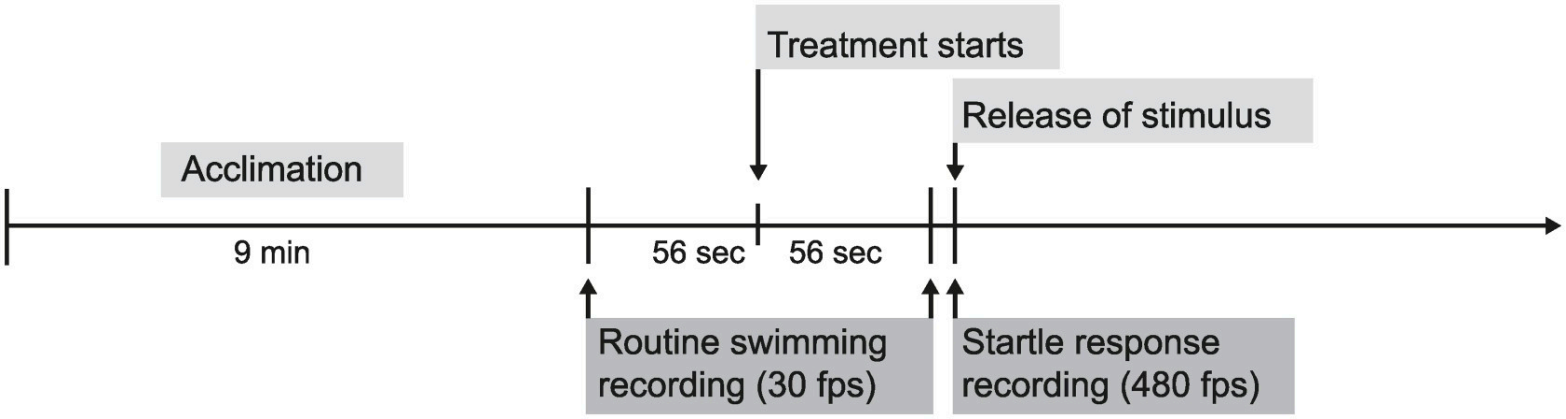
Experimental timeline. Each individual was placed in the experimental arena for nine minutes to acclimate. The routine swimming was recorded one minute before and one minute after the beginning of the sound treatment (ambient playback, 4-stroke noise playback, or ship noise playback). After recording routine swimming, a stimulus was released, and the fast-start escape response recorded.

The routine swimming of each individual was recorded one minute before and one minute after the start of the treatment to obtain an estimate of their space use and behaviour (Fig 2). Posterior analysis showed some video recordings were shorter than others, therefore analysis was standardised to 56 seconds before and 56 seconds after the start of the treatment. Speed, maximum speed and distance covered were recorded before and after the start of the treatments and the change between pre- and post-treatment calculated. Maximum speed was measured as the maximum speed reached at any point during the period of time evaluated (m/s). All variables were analysed by tracking the position of the individual every 0.5 s, which resulted in 112 data points per fish.

#### Escape response

After recording routine swimming, an escape response was elicited from the fish by the release of a tapered weight above the water surface held in place by an electromagnet (Fig 2). The drop distance of the weight was controlled by a nylon string long enough to allowed it to just break the surface of the water. To prevent a visual warning of the falling weight, the weight was released through a PVC pipe (diameter 48.5 mm) suspended above the experimental tank, with the bottom edge at 10 mm above the water level (Fig 1).

The following escape response variables were measured:

1. Responsiveness: was defined for each treatment as the proportion of individuals that responded with a sudden acceleration after being startled, out of the total number of fish.
2. Response latency (s) was measured as the time interval between the stimulus onset and the first detectable movement leading to the escape of the fish. The stimulus onset was defined as the moment the weight made contact with the surface of the water.
3. Speed (m/s) was measured as the distance covered within a fixed time (25 ms), which corresponds to the average duration of the first two flips of the tail (i.e. stages 1 and 2) [18]. This period is considered crucial for avoiding predator ambush attacks [18, 19, 35].
4. Maximum speed (m/s) was measured as the maximum speed reached at any time during the escape response.
5. Response distance (m) was determined as the total distance covered by the fish from the stimulus onset to the end of the escape response.

Additionally, the length and distance of each individual from the stimulus was recorded as a potential covariate.

### Statistical analyses

The effects of the three noise treatments on the routine swimming and escape response (excluding responsiveness) of *P*. *chrysurus* were examined using a one-factor multivariate analysis of variance (MANOVA). Pairwise, sequential Bonferroni corrected MANOVAs were used to determine the nature of differences between treatments. Response latency was affected by the distance to the drop stimulus, although it was consistent among treatments (satisfying the assumption of homogeneity of slopes). To remove this confounding influence, analysis was undertaken on the residuals of the linear relationship between response latency and distance to the drop stimulus. A canonical discriminant analysis (CDA) was used to summarise, identify and display the nature of significant differences found by MANOVA. The CDA displays graphically the strength and importance of each of the original variables, by discriminating among treatment centroids. Data were transformed to satisfy the assumptions of the test (latency, natural log; speed, power 2; response distance, power 3).

Significant differences identified by MANOVA were further examined using planned comparisons to address two independent *a priori* hypotheses: 1) vessel noise affects the routine swimming and escape response of fish (i.e., comparing ambient playback to the grouped mean of the two noise treatments); and 2) the effects of exposure to noise depend on the noise source (i.e., comparing 4-stroke noise playback vs. ship noise playback). A logistic regression was used to analyse the effect of acoustic treatment on responsiveness. Model assumptions were assessed using residual plots, all of which were satisfactory. Statistical analysis was performed in the software Statistica (Version 13.4, TIBCO) and R (Version 3.6.1, R Development Core Team, 2017).

## Results

**Fig 3:**
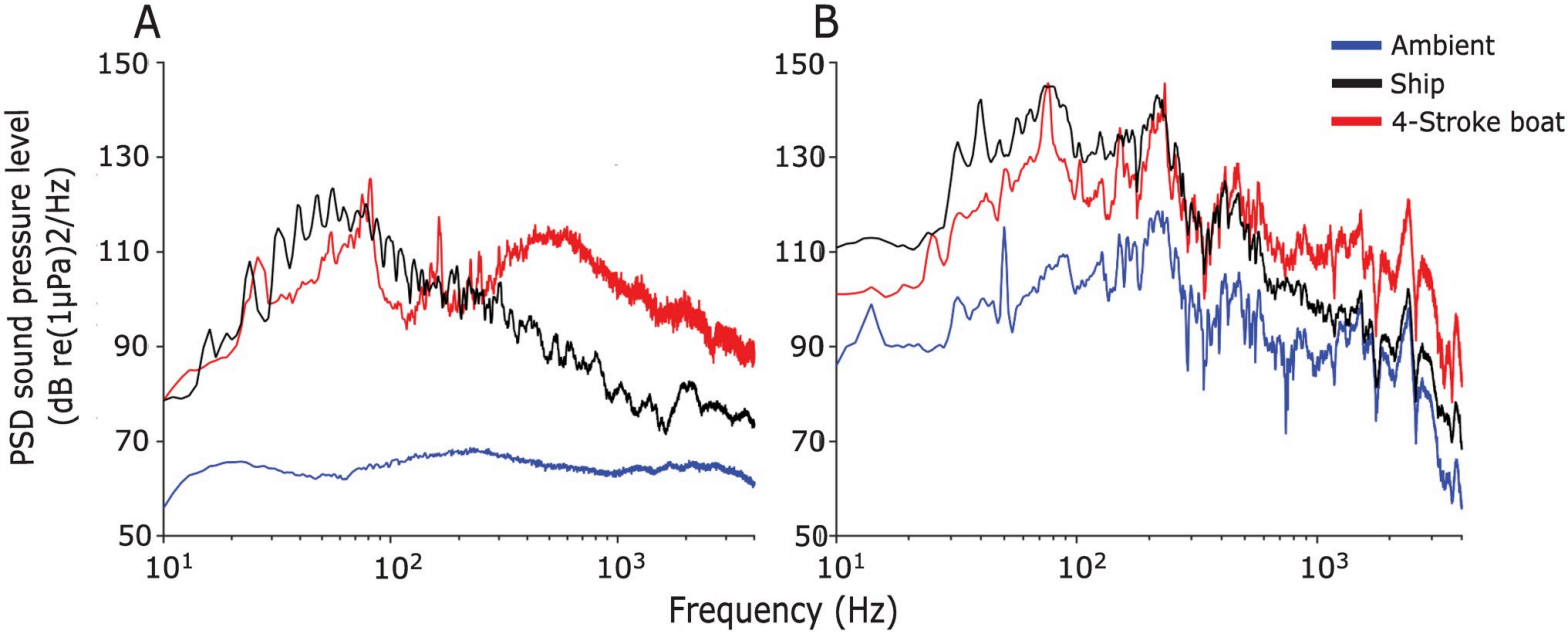
Power spectral densities (PSD) of acoustic treatments. Power spectral densities of (A) field and (B) playback of acoustic treatments. Mean PSD values were calculated from three individual samples of each noise source (Window type: 1 s Hamming, 50% overlap, frequency resolution of 1Hz). Duration of ambient, ship and 4-stroke boat playback samples for the sound analysis were approximately 30, 8 and 30 s.

Acoustic analysis indicated that playback sound pressure levels of ambient noise in the tank were lower than 4-stroke motorboat and ship noise playback levels (Fig 3a). Playback sound pressure levels of ship noise at frequencies lower than 100 Hz were higher than sound pressure levels of boat noise, while at frequencies higher than 500 Hz the sound pressure levels of boat noise were higher (Fig 3a). Playbacks differ to original recordings, probably due to near-field effects and interference cause by reflections and reverberations within the tank walls. Sound pressure in the experimental tank showed an increase in 20 dB and 30 dB for the 4-stroke motorboat and ship playbacks, respectively, compared to the field-recorded tracks (Fig 3). Despite the artefacts associated with confining sound in the tank, the relative differences between boat and ship noise across the acoustic power spectra were approximately maintained (Fig 3a vs Fig 3b). The ambient playback displayed a higher level of spectral distortion, resulting in almost 35 dB increase in sound pressure and several peaks (i.e. greater acoustic complexity) not present in the field-recorded tracks. Because the impact of the two vessel noise treatments is judged against the ambient control, this finding suggests that the results are likely to be conservative representations of the effects of vessel noise on the variables measured.

**Fig 4:**
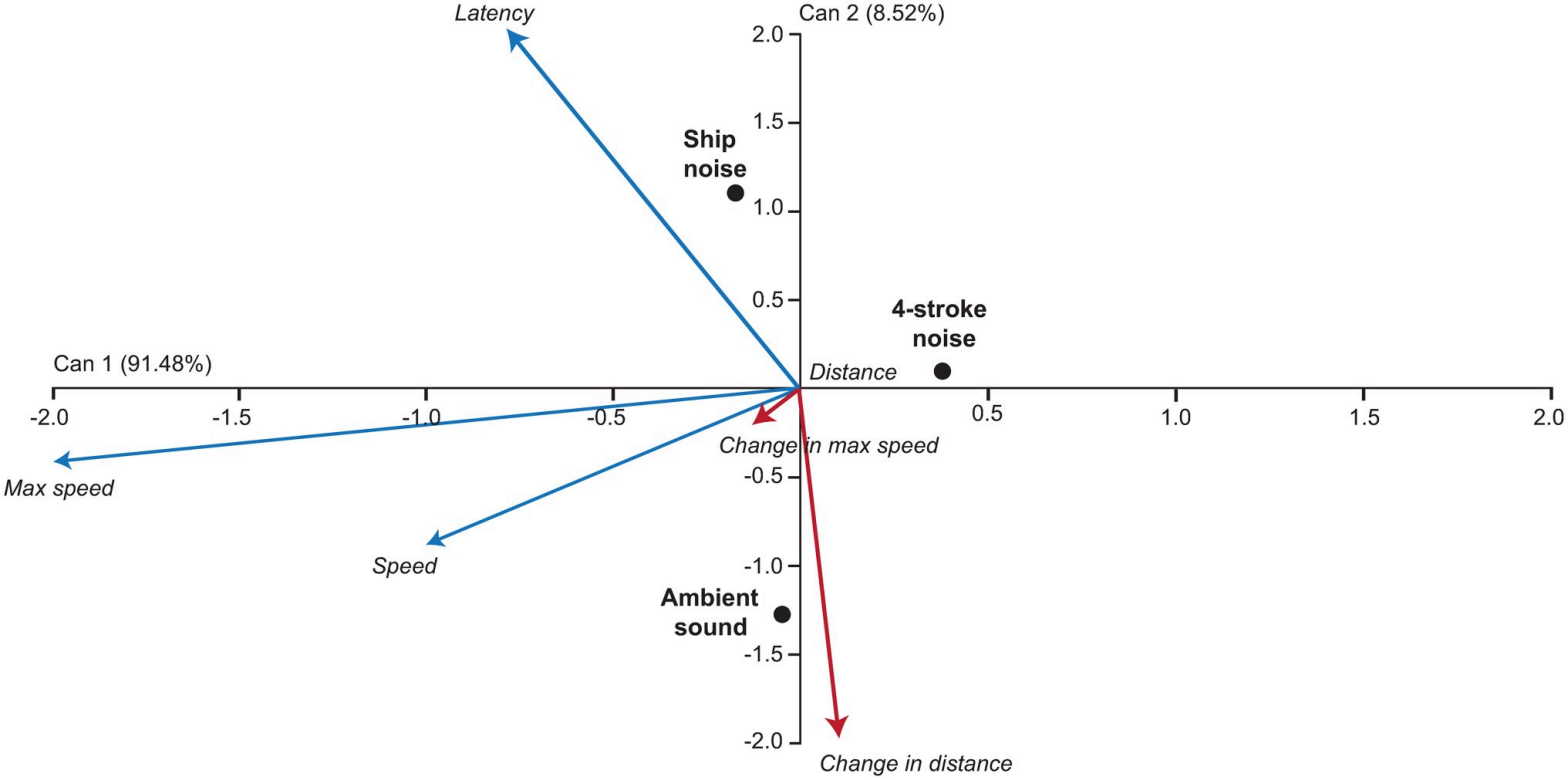
Canonical discriminant analysis. Canonical discriminant analysis displaying how the playback of noise from ships, 4-stroke powered motorboats and ambient affected the routine swimming (red line) and escape response (blue line) of juvenile *Pomacentrus chrysurus*. A canonical discriminant analysis displays the difference in routine swimming and escape response variables among acoustic treatments. The direction and importance of trends are indicated by the direction and the length of the vectors representing the original variables.

The routine swimming and escape response of *P*. *chrysurus* were significantly different among treatments (MANOVA, Pillai’s trace = 0.60, F_12,106_ = 3.84, *p* = <0.001). For the routine swimming variables, the ambient playback was significantly different from 4-stroke and ship noise playbacks (Bonferroni-corrected MANOVA, *p* = <0.001, *p* = 0.0045 respectively), while there were no differences between 4-stroke and ship noise playbacks. For the escape response variables, the three treatments were significantly different from each other (Bonferroni-corrected MANOVA, F_2,64_ = 14.77, *p* = <0.001). A CDA showed that treatments were mainly discriminated by latency and change in distance (Fig 4). Fish exposed to ambient playback and ship noise playback were differentiated along both canonical axes. Fish exposed to ship and 4-stroke motorboat noise reduced their activity, as shown by a decrease in the routine swimming variable, change in distance. Treatments were also differentiated along the axis associated with a trend in fast-start latency with fish under a ship noise playback taking longer to respond to the stimulus (i.e. increased latency) compared to fish under 4-stroke noise and the ambient playback. In addition, fish exposed to 4-stroke playback had a lower maximum speed compared to fish exposed to ship noise and ambient playbacks.

**Fig 5:**
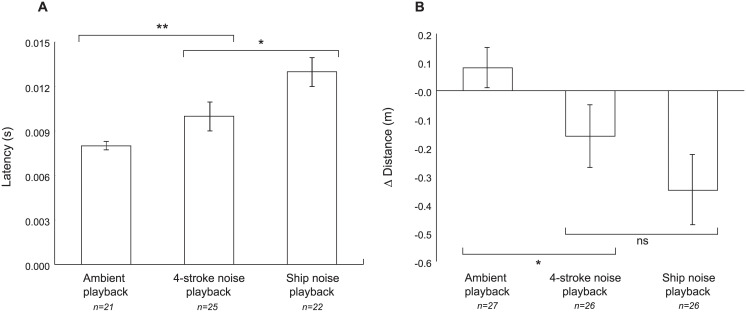
Effect of exposure to noise playbacks on routine swimming and escape response variables of *Pomacentrus chrysurus*. (A) Mean latency (± SE), (B) Change (mean ± SE) in distance covered between 56 s pre- and post-treatment. Data for latency were natural log transformed for analysis and standardised for distance to stimulus for analysis, but back-transformed covariate data are plotted. Asterisks above or below the bars represent significant differences between the planned comparisons (* = p<0.05; ns = no significant).

Univariate analyses on routine swimming and escape response confirmed the trends described by the CDA, however, there were significant differences only for latency and change in distance (Fig 5 and S7 Fig). Individuals exposed to ambient playback had almost 25% lower latency compared to individuals exposed to 4-stroke and ship noise playbacks (i.e., faster response to the drop stimulus) (Planned comparisons, SE = 0.097, *t* = -3.02, *p* = 0.004). While individuals exposed to 4-stroke playback had a shorter latency than those exposed to ship noise (Planned comparisons, SE = 0.109, *t* = -2.01, *p* = 0.048) (Fig 5A). Individuals exposed to 4-stroke and ship noise playback decreased their response distance by 15% and 30% respectively, while individuals exposed to ambient sound did not change their response distance (Planned comparisons, SE = 0.18, *t* = -2.51, *p* = 0.014). Fish exposed to boat and ship noise showed a statistically similar reduction in distance moved during routine swimming (Fig 5B, Planned comparisons, SE = 0.16, *t* = 1.17, *p* = 0.24). The proportion of individuals that responded to the stimulus was not significantly different among treatments (number displaying no reaction: ambient sound: 6; 4-strole noise: 1; ship noise: 4; S2 Table and S6 Fig).

## Discussion

Examination of the effects of noise exposure on the escape response and routine swimming of marine organisms is a relatively new area of research [14, 32, 36]. This study compared the effects on juvenile fish of noise from playbacks of 4-stroke motorboats and bulk-carrier ships, two of the most common sources of anthropogenic noise in inshore marine environments. Additionally, we used a J9 speaker, which are known to more accurately represent low frequency sounds compared to other speakers. This is noteworthy as early life-stages of many marine fishes are highly responsive to low frequency sound [37]. Our results demonstrated that exposure to vessel noise can affect the routine swimming and escape response of the whitetail damselfish. In specific, exposure to 4-stroke boat and ship noise playback decreased the total distance covered and increased the response latency of individuals compared to the ambient playback. However, the effect of ship noise was greater than the effect of 4-stroke noise indicating that different noise sources can affect fish to different degrees and in different ways. While the nature of our study question limited our methodology to a tank experiment, the results suggest that vessel noise may alter behavioural traits that have been shown to determine survival of juvenile fishes [19]. This strongly suggests that further studies are warranted on the influence of ship noise on fish behaviour on coral reefs.

The response latency of *P*. *chrysurus* was longer for individuals exposed to ship noise compared to the other acoustic treatments. Previous tank-based studies have found contradictory effects of ship noise on the response latency of fishes. For example, Simpson et al. [27] found that the response latency of European eels was 25% higher in individuals exposed to ship noise playback compared to individuals in ambient conditions, in keeping with the results of the current study. On the other hand, Voellmy et al. [28] found that sticklebacks were faster to react to a predator simulated attack (i.e. lower latency) when exposed to ship noise, while minnows did not show differences in their response latency. Studies examining the effects of 4-stroke motorboat noise have also found that this noise source can affect the escape response of fishes leading to longer escape latencies. In a field study, Simpson et al. [14] found that individuals of *P*. *ambonensis* had a higher latency, when 4-stroke motorboats where passing compared to ambient conditions, supporting our observations. In a laboratory study, McCormick et al. [36] also found that individuals of *P*. *chrysurus* exposed to 4-stroke motorboat increased their latency by 50% compared to individuals exposed to ambient playback. These studies suggest that effects of vessel noise could be species specific. In our study, the observed increase in response latency caused by exposure to ship noise and 4-stroke noise means individuals are slower to respond, therefore less likely to avoid and escape from a predator [18, 19].

In addition to the effects of noise on the fast-start response, our study provides evidence that exposure to vessel noise caused changes in space use. Analysis of routine swimming of *P*. *chrysurus* prior to the startle stimulus showed that exposure to 4-stroke motorboat and ship noise playbacks reduced the total distance moved by 15% and 40% respectively, suggesting a decrease in the activity of individuals. Only one previous study has evaluated the effects of 4-stroke on routine swimming. McCormick et al. [36] found that *P*. *chrysurus* decreased the distance moved by 20% in the first 30 seconds of exposure to 4-stroke motorboat noise, supporting our results. Routine swimming is often used as a measure of activity and boldness, related to foraging, exploratory behaviour and vigilance [19]. Previous studies have found that individuals that are more active and bolder in exploring their environment may learn information about their potential predators, have a greater appreciation of local threats and respond faster to predator strikes [38, 39]. Reduction in the time individuals allocate to these activities due to vessel exposure could theoretically increase mortality by predation [19, 40, 41], directly affect their fitness [14] and population dynamics [42].

The routine swimming and escape response of *P*. *chrysurus* were affected by 4-stroke motorboat and ship noise, however effects varied in their magnitude, with ship noise showing a greater effect. The different responses to 4-stroke motorboat and ship noise can be explain by the different acoustic properties of each treatment (e.g. rise time and frequency range). Ship noise was characterised by a rapid rise time to the highest pressure level at low frequencies (<100 Hz) that would have impacted fish without warning, while 4-stroke boat noise had slower rise time to its highest pressure level. In this portion of the frequency spectrum playback tracks correspond well with the noise levels originally recorded in the field (i.e. < 200 Hz). Moreover, low frequencies produced by ships overlap directly with the hearing ranges of recently settled damselfishes (30–1000 Hz) [2, 43, 44]. Previous studies have found that different noise sources can have differential effects on fishes. For example, McCormick et al. [30] found that exposure to 2-stroke boat noise affected boldness and activity of juvenile *Pomacentrus wardi* on patch reefs, while 4-stroke only affected activity. Moreover, while noise from 2-stroke engines prevented an effective antipredator response to alarm odours, no such effect occurred in response to noise from 4-stroke powered boats. Our findings suggest that ship noise is likely to have a similar, if not greater impact on risk assessment than noise from 4-stroke engines, compromising in theory their anti-predator behavioural in their natural environment [14, 19].

Complementary laboratory and field-based studies have been recognised as essential for the understanding of noise pollution and its effects on marine organisms [14, 45, 46]. Our experiment was conducted in a laboratory set-up that allowed for a detail examination of behavioural traits of individuals placed under identical acoustic conditions [23]. However, there are acoustic limitations to tank-based experiments [24]. The playbacks used in our study differed from the original recordings, in particular the ambient playback, therefore is possible that the effects of the acoustic treatments are under or overestimated. Still our results showed different effects from each of the acoustic treatments and suggest that some information was not lost [45]. Our study contributes to the growing body of literature documenting the effects of anthropogenic noise on reef fish and represents an important stepping stone in the understanding of ship noise pollution and its effects on reef fishes. Further field based studies are required to complement our findings and to determine the effects of long-term noise exposure and the capacity of fishes to acclimate or habituate to this disturbance.

## Supporting information

S1 Fig Grid design to investigate the sound pressure and particle motion in experimental tank. Colour and letters indicate position where posterior sound recordings were made within the grid.
(PNG)

S2 Fig Particle acceleration measurements from tank playback grid experiment. Colour plots represent longitudinal grid position in relation to proximity to the speaker (blue—1^st^, black—2^nd^, red—3^rd^, purple—4^th^). Letters represent transverse grid position (A—centre, B—right, C—left).
(PNG)

S3 Fig Sound pressure measurements from tank playback grid experiment. Colour plots represent longitudinal grid position in relation to proximity to the speaker (blue—1^st^, black—2^nd^, red—3^rd^, purple—4^th^). Letters represent transverse grid position (A—centre, B—right, C—left).
(PNG)

S4 Fig Particle acceleration measurements from tank playback experiment. Colour plots represent playback samples (n = 3 separate sound tracks).
(PNG)

S5 Fig Sound pressure measurements from tank playback experiment. Colour plots represent playback samples (n = 3 separate sound tracks).
(PNG)

S6 Fig Proportion of individuals that performed an escape response among treatments. (JPG)

S7 Fig Effect of noise exposure on routine swimming and escape response variables *Pomacentrus chrysurus*. (A) Speed (mean ± SE), (B) Maximum speed (mean ± SE), (C) Distance (mean ± SE), (D) Change (mean ± SE) in speed and (E) change (mean ± SE) in maximum speed between pre- and post-treatment. Back-transformed data are plotted.
(EPS)

S1 Table Details of ambient, 4-stroke boats and ship recordings used in playback experiments. (DOCX)

S2 Table Summary of logistic regression comparing the number of responsive individuals among acoustic treatments. (DOCX)

S3 Table Tukey test for responsiveness. (DOCX)
