# Supplementary figures and images for: Vessel noise affects routine swimming and escape response of a coral reef fish
Source: PLoS One. 2020 Jul 23;15(7):e0235742. doi: 10.1371/journal.pone.0235742 (PMC7377389; doi:10.1371/journal.pone.0235742)

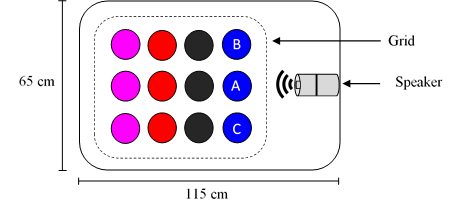

Supplement: S1 Fig — Colour and letters indicate position where posterior sound recordings were made within the grid. (PNG) [file pone.0235742.s001.png]

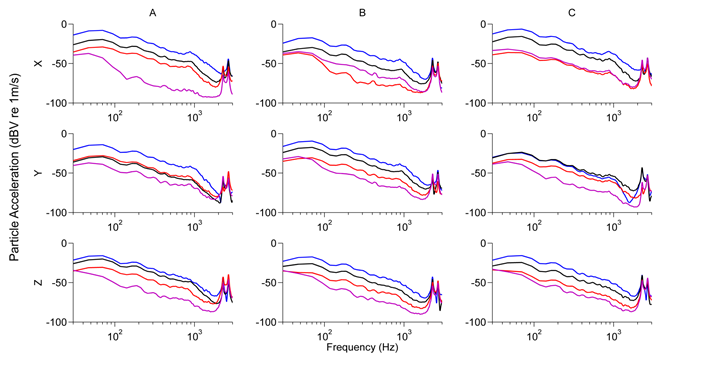

Supplement: S2 Fig — Colour plots represent longitudinal grid position in relation to proximity to the speaker (blue—1st, black—2nd, red—3rd, purple—4th). Letters represent transverse grid position (A—centre, B—right, C—left). (PNG) [file pone.0235742.s002.png]

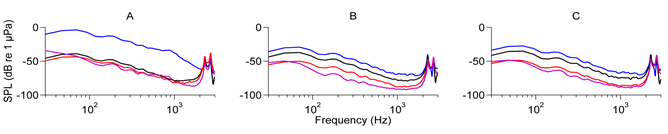

Supplement: S3 Fig — Colour plots represent longitudinal grid position in relation to proximity to the speaker (blue—1st, black—2nd, red—3rd, purple—4th). Letters represent transverse grid position (A—centre, B—right, C—left). (PNG) [file pone.0235742.s003.png]

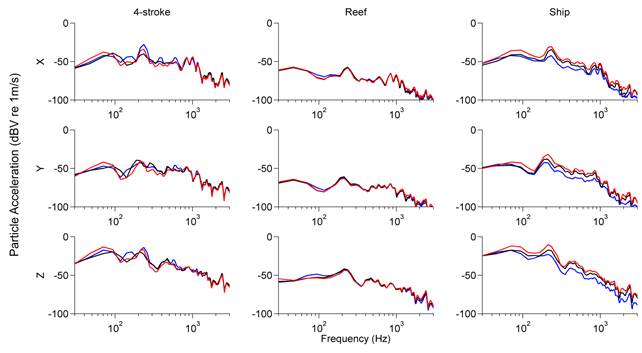

Supplement: S4 Fig — Colour plots represent playback samples (n = 3 separate sound tracks). (PNG) [file pone.0235742.s004.png]

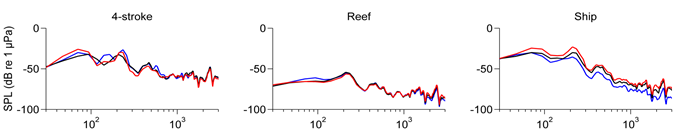

Supplement: S5 Fig — Colour plots represent playback samples (n = 3 separate sound tracks). (PNG) [file pone.0235742.s005.png]

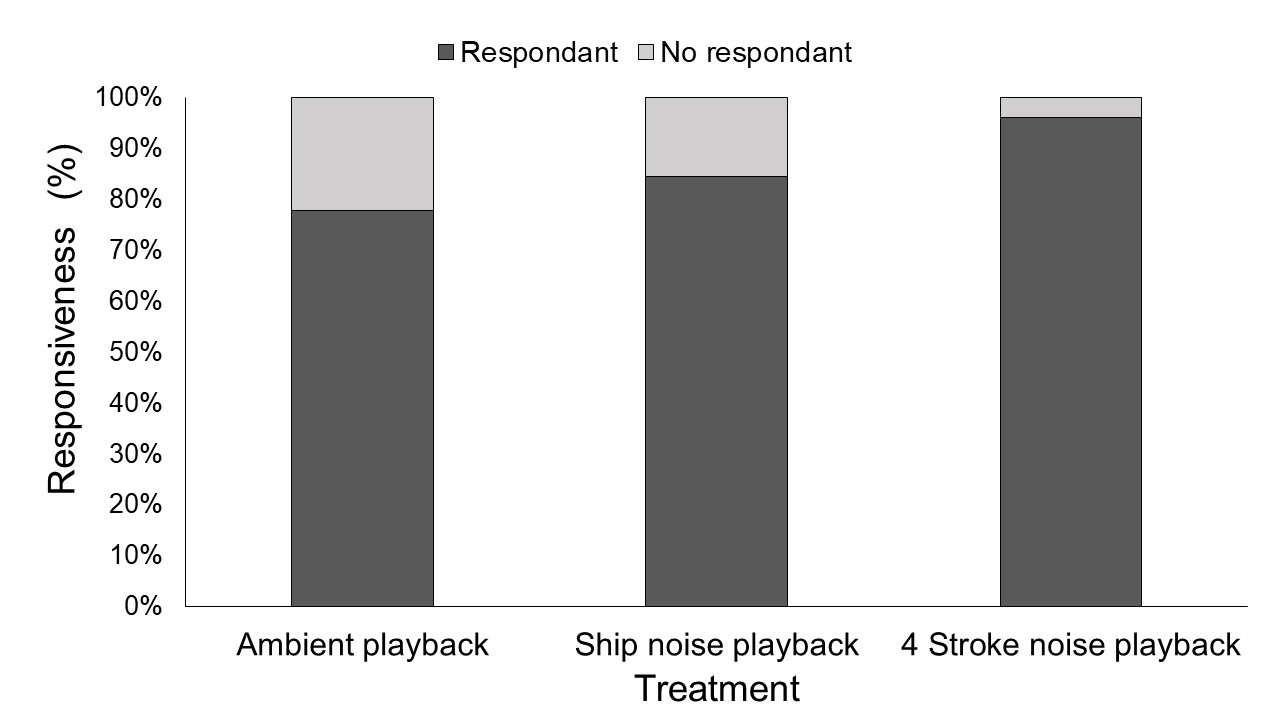

Supplement: S6 Fig — (JPG) [file pone.0235742.s006.jpg]
