# Supplementary material for: Vessel noise affects routine swimming and escape response of a coral reef fish
Source: PLoS One. 2020 Jul 23;15(7):e0235742. doi: 10.1371/journal.pone.0235742 (PMC7377389; doi:10.1371/journal.pone.0235742)
Supplement: S1 Table — (DOCX) [file pone.0235742.s008.docx]

**S1 Table. Details of ambient, 4-stroke boats and ship recordings used in playback experiments.**

| **Treatment** | **Replicate** | **Location** | **Distance to receiver** | **Receiver depth** | **Vessel** | **Tonnage** | **Engine type** | **Engine power (kW)** |
| --- | --- | --- | --- | --- | --- | --- | --- | --- |
| Ambient | 1 | Lagoon |  | 6 - 9 m |  |  |  |  |
|  | 2 | Palfrey inner |  | 6 - 9 m |  |  |  |  |
|  | 3 | Inner Bird |  | 6 - 9 m |  |  |  |  |
| Ship | 1 | Big V outer | 1.9-2.5 km | 16-19 m | RTM Twarra | 53988 | MAN-B&W Diesel | 13,501 |
|  | 2 | Eagle | 1.9-3.2 km | 17-20 m | RTM Gladstone | 53988 | MAN-B&W Diesel | 13,501 |
|  | 3 | Eagle | 2.0-3.0 km | 17-20 m | RTM Piiramu | 53988 | MAN-B&W Diesel | 13,501 |
| 4-stroke | 1 | Lagoon middle ST | 1 - 25 m | 2 m | Research boat |  | Yamaha 4-stroke 30 hp | 22 |
|  | 2 | Lagoon middle ST | 1 - 25 m | 2 m | Research boat |  | Yamaha 4-stroke 30 hp | 22 |
|  | 3 | Lagoon - close to reef | 1 - 25 m | 2 m | Research boat |  | Yamaha 4-stroke 30 hp | 22 |
