# Supplementary material for: Vessel noise affects routine swimming and escape response of a coral reef fish
Source: PLoS One. 2020 Jul 23;15(7):e0235742. doi: 10.1371/journal.pone.0235742 (PMC7377389; doi:10.1371/journal.pone.0235742)
Supplement: S2 Table — (DOCX) [file pone.0235742.s009.docx]

**S2 Table. Summary of logistic regression comparing the number of responsive individuals among acoustic treatments.**

| **Predictor variable** | **Estimate** | **SE** | **Z value** | **P value** |
| --- | --- | --- | --- | --- |
| Ambient playback | 1.2528 | 0.4629 | 2.706 | 0.0068 |
| Ship noise playback | 0.4520 | 0.7140 | 0.633 | 0.5267 |
| 4-Stroke noise playback | 1.9661 | 1.1196 | 1.756 | 0.0791 |
