# Supplementary material for: Vessel noise affects routine swimming and escape response of a coral reef fish
Source: PLoS One. 2020 Jul 23;15(7):e0235742. doi: 10.1371/journal.pone.0235742 (PMC7377389; doi:10.1371/journal.pone.0235742)
Supplement: S3 Table — (DOCX) [file pone.0235742.s010.docx]

**S3 Table. Tukey test for responsiveness**

| **Contrast** | **Estimate** | **SE** | **Z value** | **P value** |
| --- | --- | --- | --- | --- |
| Ambient – ship | -0.452 | 0.714 | -0.633 | 0.8019 |
| Ambient – 4 stroke | -1.966 | 1.120 | -1.756 | 0.1846 |
| Ship- 4 stroke | 1.514 | 1.155 | -1.311 | 0.3892 |
